# Congenital Dyserythropoietic Anemia Type II: A Case Report

**DOI:** 10.7759/cureus.27933

**Published:** 2022-08-12

**Authors:** Muhammad Mujeeb Hassan, Azka A Mirza, Rafay Zaidi, Moeena Malik, Maham Javaid

**Affiliations:** 1 Internal Medicine, Birmingham Heartlands Hospital, Birmingham, GBR; 2 Medicine, Benazir Bhutto Hospital, Rawalpindi, PAK; 3 Internal Medicine, Al-Nafees Medical College, Islamabad, PAK; 4 Internal Medicine, Benazir Bhutto Hospital, Rawalpindi, PAK; 5 Internal Medicine, Allama Iqbal Medical College, Lahore, PAK

**Keywords:** severe anemia, multi-disciplinary team, rare genetic diseases, bone marrow biopsy, type ii congenital dyserythropoietic anemia

## Abstract

Congenital dyserythropoietic anemia (CDA) type 2 is a rare genetic disease that presents with mild to severe anemia. The rare occurrence may be a reason why CDAs are often misdiagnosed since the morphological abnormalities and the clinical features are commonly found in other clinically-related anemias. We report a case of a 17-year-old male who presented in a tertiary care government hospital, with a history of lethargy, abdominal pain, abdominal fullness, and failure to thrive. Bone marrow biopsy reported the uncommon diagnosis of CDA type 2, the Ham test was also positive. The management included a multi-disciplinary approach alongside counseling of the family.

## Introduction

Congenital dyserythropoietic anemia (CDA) type 2 is an inherited blood disorder rarely seen in clinical settings. It is usually caused due to changes in the *SEC23B* gene. CDA type 2 is commonly diagnosed during adolescence or early childhood and presents with mild to severe anemia. Patients may also develop jaundice, hepatosplenomegaly, and gallstones. There are three classical types of CDA, and six types in total, defined by bone marrow morphology. Among these, CDA type 2 is the most common. CDAs are often misdiagnosed since the morphological abnormalities and the clinical features can also be found in other clinically-related anemias. This case report discusses the case of a 17-year-old male who presented with a history of lethargy, abdominal pain, abdominal fullness, and failure to thrive, for the past three years. Bone marrow biopsy reported the diagnosis of CDA type 2, the Ham test was also positive.

## Case presentation

A 17-year-old male presented to the outpatient department of Benazir Bhutto Hospital, Rawalpindi, after being referred from a district headquarters hospital, where he had presented with complaints of lethargy, abdominal pain, abdominal fullness, and failure to thrive. Baseline investigations suggested that the patient had pancytopenia, neutropenia, and a low mean corpuscle volume of red blood cells. On further inquiry, it was identified that the patient had the above-mentioned symptoms for the past three years. He also complained of shortness of breath after minimal exertion along with dizziness; however, there was no orthopnea or paroxysmal nocturnal dyspnea. He had been previously treated with antibiotics and antifungals for multiple chest infections and oral thrush. The patient’s parents had a consanguineous marriage and reported the presence of unknown blood disorders in the family, which had led to reduced life expectancy for some members of the family.

At presentation, physical examination revealed significant pallor. The patient was fully conscious and oriented. His heart rate was 110 bpm, blood pressure was 90/60 mmHg, respiratory rate was 18/min, peripheral capillary oxygen saturation (Spo2) was 95% on room air, and he was afebrile. The patient weighed only 30 kg while his height was 96 cm. Examination of the abdomen revealed marked spleen enlargement; however, there was no evidence of jaundice or lymphadenopathy. A complete blood count (CBC) was obtained, which showed a decrease in all cell lines as shown in Table 1. The CBC, which showed pancytopenia with all cell lines significantly decreased. White blood cell count was 400 uL (neutrophils (600 uL), lymphocytes (600 uL), and mixed white blood cells (200 uL)), hemoglobin was 4.5 g/dL with a mean corpuscle volume of 58.9 fL, and platelets were only 126,000 uL. The patient was admitted for further investigation.

**Table 1:** Complete blood count HCT: hematocrit; MCV: mean corpuscular volume; MCHC: mean corpuscular hemoglobin concentration; RDW: red cell distribution width; PDW: platelet distribution width; MPV: mean platelet volume

Complete blood Count	Results	Unit	Normal Range
WBC Count	1.4	10^3 /uL	4.0 - 11.0
RBC Count	2.87	10^6/ uL	3.50 - 5.50
Hemoglobin	4.5	g/dL	12.0 - 16.0
HCT	16.9	%	35.0 - 50.0
MCV	58.9	fL	76.0 - 96.0
MCH	15.6	pg	26.0 - 32.0
MCHC	26.6	g/dL	32.0 - 36.0
Platelets	126	10^3/ uL	150.0 - 450.0
Lymphocytes	0.6	10^3/ uL	-
Mixed	0.2	10^3/ uL	-
Neutrophils	0.6	10^3/ uL	-
RDW	27.4	fL	42.0 - 47.0
PDW	15.5	fL	-
MPV	7.7	fL	-

Blood films showed a dimorphic blood picture with significant hypochromia and microcytosis (Table 2). Reticulocyte count was only 1.9%. Liver function tests were within the normal range except for a prothrombin time of 19 seconds with the control being 14 seconds. Renal function tests were within the normal range. Ultrasound of the abdomen and pelvis revealed the liver span as 13.4 cm, portal vein diameter was 12 mm with a velocity of 14 cm/s, and a massively enlarged spleen with a splenic vein diameter of 7.3 mm. However, no varices were noted. Upper gastrointestinal endoscopy was also performed due to portal hypertension and revealed mild gastritis. The causes of splenomegaly were investigated with the help of an infection screen, which included blood cultures, viral infections, malaria, and leishmaniasis; however, the results for all of the above were negative. Furthermore, written consent was obtained for a bone marrow biopsy, which was performed on the posterior superior iliac spine. Biopsy results (Table 3) revealed CDA type 2 and the Ham test was also positive.

**Table 2:** Peripheral blood smear

Morphology	Result
Anisocytosis	+
Poikilocytosis	+
Microcytosis	++
Hypochromia	++
Macrocytosis	+
Reticulocytes	1.9 %
Comments	Dimorphic blood picture

**Table 3:** Bone marrow biopsy findings suggestive of congenital dyserythropoietic anemia M:E ratio: myeloid to erythroid ratio

Finding	Description
Site of biopsy	Posterior superior iliac spine
Cellularity	Hypercellular fragments and trails
Erythropoiesis	Hypercellular, mild megaloblastic, normoblasts, dyserythropoietic with multinucleate forms
Myelopoiesis	Hyperplastic with normal maturation
M:E ratio	<1
Megakaryocytes	Increased
Lymphocytes	Normal
Plasma cells	Normal
Histiocytes	Normal
Abnormal cells	Nil
Blast cells	Nil
Extramedullary cells	Nil
Parasites	Nil
Iron	Absent
Comments	Congenital dyserythropoietic anemia type 2 (CDA type 2); Ham test is positive

Management of this patient required a multi-disciplinary approach. The patient was educated about the disease, and an appointment was booked with the genetic counselor to further help the family. The opinion of a hematologist was sought and frequent blood transfusions were advised, after every two to three weeks. The patient had to be observed for iron overload and potentially be started on chelation therapy. The patient was also counseled about the option of curative splenectomy or cholecystectomy in the future to avoid complications as a result of hemolysis.

## Discussion

CDA type 2 is the most commonly diagnosed variant of the CDAs. More than 200 cases have been identified but there have been no follow-up reports on the lifetime evolution of the disease [1]. The estimated number of people with this disease may be between 1-300 in the United States [2]. It usually presents in childhood and has a clinical picture similar to chronic anemia of mild to moderate degree; splenomegaly and intermittent or persistent jaundice are also commonly present [3]. Recent studies have indicated that CDA type 2 is caused by a defect disturbing Golgi processing in erythroblasts. Linkage analysis has located a candidate region on chromosome 20, termed the CDAN2 locus, in a majority of patients diagnosed with CDA type 2; however, the aberrant gene has not so far been elucidated [4]. Diagnosis is confirmed after bone marrow biopsy and the Ham test provides additional evidence. The prevalence of CDA type 2 is probably underestimated since its clinical spectrum has not yet been well-defined; therefore, it is often misdiagnosed with more frequent clinically-related anemias including thalassemia syndromes and hereditary sideroblastic anemia [5]. The careful management of iron overload is required in patients diagnosed with CDA type 2 requiring blood transfusions. It is suggested that the most favorable outcomes are achieved when CDA type 2 patients are managed with a holistic and multidisciplinary approach [6]. One of the main goals should be to provide guidance and support to the patient and their family along with adequate information about future options that may help the patient. These options may involve splenectomy and cholecystectomy. This goal is achieved by an interprofessional team that includes primary care physicians, hematologists, general surgeons, specialty-trained nurses, and psychologists. Medical care should be initiated with blood transfusions every two to three weeks and close observation for the development of iron overload in the patient [7].

## Conclusions

CDA type 2 is a rare genetic disorder that is commonly misdiagnosed due to its presentation being fairly similar to other commonly observed anemias. The management of CDA requires a multi-disciplinary approach that involves hematologists, geneticists, pediatricians, surgeons, and counselors. It is also important to closely monitor all patients diagnosed with CDA receiving blood transfusions due to the high chances of iron overload. Furthermore, due to the hereditary nature of the disorder, we also recommend the evaluation of the siblings and close family members of a diagnosed patient so that the monitoring of hemoglobin and iron levels along with required treatment can begin as soon as possible for the affected patients. At present, further research is mandatory to understand the lifetime evolution of this disease.
